# Employer support for employees returning to work from sick leave – evidence for Europe

**DOI:** 10.1093/eurpub/ckac129.095

**Published:** 2022-10-25

**Authors:** T Leoni

**Affiliations:** University of Applied Sciences Wiener Neustadt, Wiener Neustadt, Austria

## Abstract

**Background:**

Employers play an important role in facilitating the return-to-work (RTW) of employees after a prolonged sick leave. The involvement of employers in RTW efforts is however largely unexplored in an international comparative context. This paper provides evidence on the diffusion of procedures to support RTW after sick leave in European workplaces and discusses different policy approaches to involve employers in RTW.

**Methods:**

Employer activities are examined using microdata from the three waves of the European Survey on New and Emerging Risks (ESENER), collected in 2009, 2014 and 2019 (N = 47,425). ESENER is a representative company survey, conducted on behalf of the European Agency for Safety and Health at Work (EU-OSHA). The outcome of interest is information on the existence of a procedure to support employees’ RTW after a long sickness absence. The analysis is carried out using logistic models, comparing countries and welfare state regimes.

**Results:**

Overall, 71.8% percent of workplaces with more than 50 employees have procedures to support RTW. Employer support is most common in Nordic and Anglo-Saxon countries (OR = 3.2, 95% CI 2.58-4.05 and OR = 8.9, 95% CI 5.41-14.68 respectively, compared to Continental countries and accounting for firm size and sector of activity). In Southern and Eastern Europe, the diffusion of RTW support is lowest (OR = 0.3, 95% CI 0.26-0.37 and OR = 0.2, 95% CI 0.17-0.24). With the exception of Nordic and Anglo-Saxon countries, smaller establishments are significantly less likely to have procedures in place than larger ones. The observed patterns are stable over time.

**Conclusions:**

Employer support for RTW varies greatly across countries and welfare state regimes, indicating that institutional settings and policies matter for employer involvement in RTW, particularly in smaller workplaces. Countries with high levels of RTW support display different combinations of legal obligations and/or incentives for employers to support RTW.

**Key messages:**

• There are large differences in employer support for RTW after sick leave across Europe.

• High support levels are associated with policies combining obligations and/or incentives for employers.

